# AMPA and angiotensin type 1 receptors are necessary for hemorrhage-induced vasopressin secretion

**DOI:** 10.1590/1414-431X2021e11635

**Published:** 2022-02-04

**Authors:** R.C. Dos-Santos, T. Vilhena-Franco, L.C. Reis, L.L.K. Elias, J. Antunes-Rodrigues, A.S. Mecawi

**Affiliations:** 1 Departamento de Fisiologia, Faculdade de Medicina de Ribeirão Preto, Universidade de São Paulo, Ribeirão Preto, SP, Brasil; 2 Departamento de Ciências Fisiológicas, Universidade Federal Rural do Rio de Janeiro, Seropédica, RJ, Brasil; 3 Laboratório de Neuroendocrinologia, Departamento de Biofísica, Escola Paulista de Medicina Universidade Federal de São Paulo, São Paulo, SP, Brasil

**Keywords:** Vasopressin, Hemorrhage, AMPA, NMDA, Angiotensin receptor type 1

## Abstract

Hypovolemia induced by hemorrhage is a common clinical complication, which stimulates vasopressin (AVP) secretion by the neurohypophysis in order to retain body water and maintain blood pressure. To evaluate the role of brain L-glutamate and angiotensin II on AVP secretion induced by hypovolemia we induced hemorrhage (∼25% of blood volume) after intracerebroventricular (*icv*) administration of AP5, NBQX, or losartan, which are NMDA, AMPA, and AT1 receptor antagonists, respectively. Hemorrhage significantly increased plasma AVP levels in all groups. The *icv* injection of AP5 did not change AVP secretion in response to hemorrhage. Conversely, *icv* administration of both NBQX and losartan significantly decreased plasma AVP levels after hemorrhage. Therefore, the blockade of AMPA and AT1 receptors impaired AVP secretion in response to hemorrhage, suggesting that L-glutamate and angiotensin II acted in these receptors to increase AVP secretion in response to hemorrhage-induced hypovolemia.

## Introduction

Hemorrhage is common in clinical settings and can have several causes such as injuries, diseases, surgeries, childbirth, gastrointestinal pathologies, among others (1). Severe bleeding can cause hypovolemic shock and, consequently, death (1). In response to hypovolemia and/or hypotension, magnocellular neurons in the paraventricular (PVN) and supraoptic (SON) hypothalamic nuclei secrete arginine vasopressin (AVP) through the neurohypophysis into the blood (2,3). AVP then acts on V_2_ receptors in the kidney to increase water reabsorption and, consequently, decrease urinary water loss. Concomitantly, AVP also acts on V_1_ receptors in blood vessels and exerts its vasoconstrictor effects (3). Thus, AVP secretion plays an important role in survival after hemorrhage.

AVP secretion is dependent on the activity of magnocellular neurons, which is controlled by a complex neural circuit that integrates the information regarding blood pressure and extracellular fluid volume and osmolality (3). The information concerning blood volume and arterial pressure is transmitted to the brain from baroreceptors and volume receptors, which send afferent signals to the nucleus of the solitary tract (4). In turn, this nucleus sends projections to the magnocellular neurons in the PVN and SON that respond either by increasing or decreasing AVP release as necessary to maintain the homeostasis of the body water and arterial pressure (3). Osmorreceptor neurons at the circumventricular organs respond to changes in plasma osmolality and sodium levels. These nuclei project to the PVN and SON increasing the activity of magnocellular neurons and, consequently, increasing AVP secretion in response to hyperosmolality. Furthermore, the magnocellular neurons are also intrinsically osmossensitive, directly responding to changes in extracellular osmolality to modulate AVP production and secretion (3).

The afferent input to the magnocellular neurons is influenced by a series of mediators, among which are angiotensin II (ANG II) and L-glutamate (5). Intracerebroventricular (*icv*) losartan administration blocks the ANG II-induced AVP release, indicating that AT1 receptors are responsible for this response (6). Additionally, studies in brain slices demonstrated that ANG II depolarizes magnocellular neurons in the PVN (7) and SON (8). Mice with knockdown of AT1 receptors in AVP neurons exhibited decreased AVP secretion in response to hypertonic saline loading or icv ANG II administration (9).

Ionotropic glutamate receptors, such as α‐amino‐3‐hydroxy-5‐methylisoxazole-4‐propionic acid (AMPA) and N-methyl-D-aspartic acid (NMDA), have been previously shown to mediate AVP secretion (10,11). Administration of L-glutamate directly into the SON induces AVP release, an effect that may be blocked by AMPA receptor antagonists in basal conditions (10). In hypothalamus-neurohypophysis explants, AMPA receptor antagonists block the AVP secretion in response to osmotic stimulation (11), whilst metabotropic glutamate receptors did not influence AVP release (12). We have recently shown that L-glutamate stimulates the secretion of AVP through NMDA receptors after hypertonic extracellular volume expansion (EVE) (13). The participation of different ionotropic L-glutamate receptors in hemorrhage-induced AVP release is not known. Thus, the present study aimed to evaluate the participation of L-glutamate AMPA and NMDA receptors and ANG II AT1 receptors in the brain on hemorrhage-induced AVP secretion.

## Material and Methods

### Animals

All procedures were approved by the Ethics Committee of the Ribeirão Preto Medical School, University of São Paulo (#36/2010) and were conducted in accordance with the current legislation. Male Wistar rats (∼300 g) were obtained from the animal facility of Ribeirão Preto Medical School and maintained in collective cages (41×34×16 cm, four rats per cage) in a room with controlled temperature (23±2°C) and 12-h light/dark cycle (lights on from 6 am to 6 pm) with free access to filtered tap water and standard rat chow.

### *icv* cannula implantation and drug microinjection

The animals were anesthetized with 2.5% 2,2,2-tribromoethanol (1 mL/100 g body weight, intraperitoneal). When in anesthetic plane, animals were placed in a stereotaxic apparatus. Next, a cannula (stainless steel; 0.7 mm external diameter, 0.4 mm internal diameter, and 10 mm long) was inserted into the right lateral ventricle at the coordinates: 0.5 mm posterior to bregma, 1.4 mm lateral from the middle line, 3.6 mm down from the skull, as previously described (13). All animals received a prophylactic dose of antibiotic (50,000 units of penicillin G, intramuscular). After surgery, the animals were placed in individual cages for a 7-day recovery period, during which they were handled daily in order to decrease the influence of handling stress. Then, 20 min before hemorrhage, animals were submitted to *icv* microinjection of 5 μL/rat of vehicle (0.9% saline solution), NBQX (#1044, Tocris Cookson Ltd., United Kingdom), AP5 (#A8054, Sigma Chemical Co., USA), or losartan (Merck Sharp & Dohme, USA). Both NBQX and AP5 were used at doses of 10 and 30 nmol/5 μL per rat, as previously described (13). Losartan was used at a dose of 100 nmol/5 μL per rat (14).

### Femoral artery cannulation and hemorrhage procedure

The hemorrhage procedure was carried out as previously described (2). A polyethylene cannula (PE-10 connected to PE-50, Intramedic; Becton-Dickinson, USA) was inserted into the right femoral artery and externalized in the dorsal cervical region. Then, the cannula was flushed with isotonic saline with heparin (100 IU/mL Liquemine; Roche, Switzerland) to prevent obstruction. Twenty-four hours after femoral artery cannula implantation, isotonic saline with heparin (150 IU in 150 µL) was administered through the femoral catheter to prevent blood clotting; then the blood was removed through the femoral artery (15 mL/kg body weight, ∼25% of blood volume in 1 min) 20 min after the *icv* microinjection. In the control group, the same procedures were carried out without blood removal.

### Hormone assessment

The animals were euthanized by decapitation 5 min after hemorrhage, and the blood was collected from the trunk in polypropylene tubes kept on ice, containing anticoagulant (15 IU of heparin/mL of blood). The plasma was separated by centrifugation at 1600 *g* for 20 min at 4°C and then stored at -20°C until extraction. Plasma AVP concentration was measured by specific radioimmunoassay as previously described (13). Assay sensitivity and intra- and inter-assay coefficients of variation were 0.7 pg/mL, 7.6, and 12%, respectively.

### Statistical analysis

GraphPad Prism v. 7.0 software (USA) was used for all statistical analyses. A two-way analysis of variance followed by Bonferroni *post hoc* test was used to compare groups. All data are reported as means±SE.

## Results

**Figure 1:**
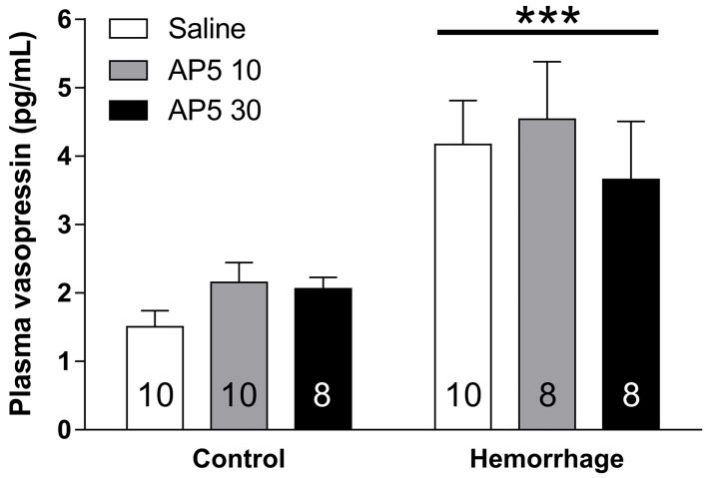
The intracerebroventricular administration of AP5 did not affect plasma arginine vasopressin (AVP) after hemorrhage. This result suggests that L-glutamate NMDA receptors are not necessary for hemorrhage-induced AVP secretion. Data are reported as means±SE. ***F_(1,48)_=23.73, P<0.001 compared to control (no hemorrhage) (ANOVA). Numbers in columns indicate the number of animals/group.

In order to assess the influence of NMDA receptors on the response to hemorrhage, we microinjected AP5, a NMDA antagonist, 20 min prior to hemorrhage *icv*. Hemorrhage significantly increased plasma AVP (F_(1,48)_=23.73, P<0.001) while *icv* administration of AP5 did not change AVP plasma levels (F_(2,48)_=0.54, P=0.59), with no interaction between the two factors (F_(2,48)_=0.48, P=0.62), suggesting that the NMDA receptors were not involved in the AVP secretion in response to hemorrhage (Figure 1).

**Figure 2:**
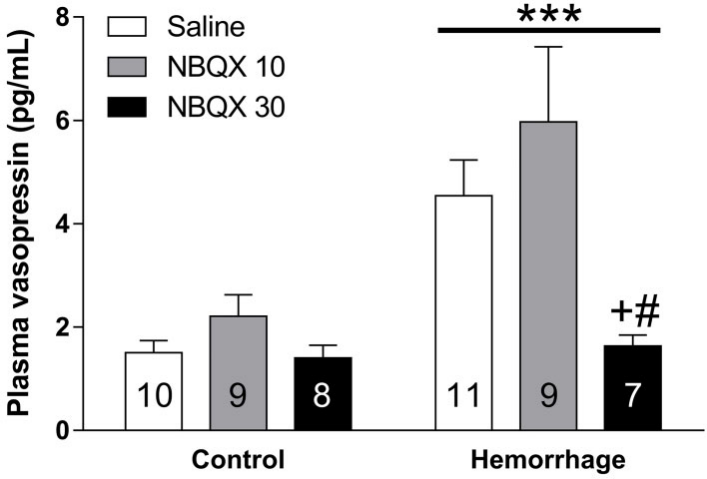
Intracerebroventricular administration of NBQX reverses the effect of hemorrhage on plasma arginine vasopressin (AVP). Thus, L-glutamate AMPA receptor signaling seems to be involved in the hemorrhage-induced AVP secretion. Data are reported as means±SE. ***F_(1,48)_=15.24, P<0.001 compared to control; ^+^P=0.02 *vs* hemorrhage-saline; ^#^P<0.001 *vs* hemorrhage-NBQX 10 (ANOVA). Numbers in columns indicate the number of animals/group.

Next, we used the NBQX, an AMPA receptor antagonist, to evaluate the participation of these receptors in AVP secretion after hemorrhage. Both hemorrhage (F_(1,48)_=15.24, P<0.001) and *icv* NBQX administration (F_(2,48)_=5.68, P=0.006) significantly changed plasma AVP levels, and the interaction between the two factors tended toward statistical significance (F_(2,48]_=2.92, P=0.06). The *post hoc* analysis showed that NBQX *per se* did not change AVP secretion in control animals, whilst after hemorrhage the highest dose of NBQX significantly lowered plasma AVP levels compared to saline (P=0.049) or low dose of NBQX (P=0.001) (Figure 2). These data showed that the blockade of AMPA receptors blunted hemorrhage-induced AVP secretion.

**Figure 3:**
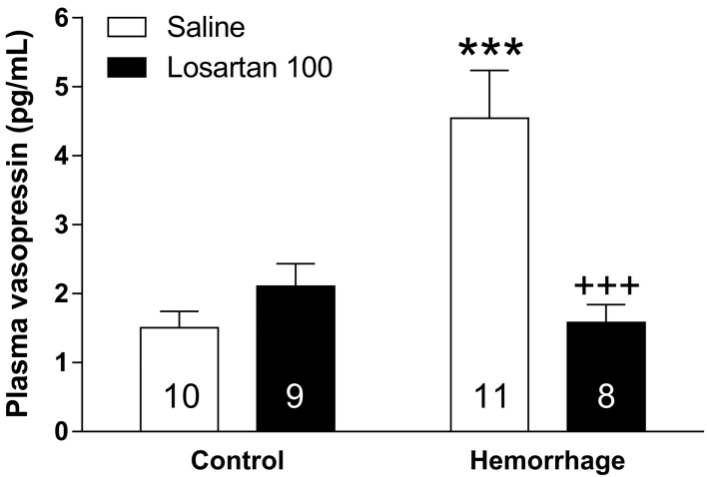
Losartan administration reverses the effects of hemorrhage on arginine vasopressin (AVP) secretion. This result indicates that angiotensin II AT1 receptor signaling is necessary for hemorrhage-induced AVP secretion. Data are reported as means±SE. ***P<0.001 *vs* control-saline, ^+++^P<0.001 *vs* hemorrhage-saline (ANOVA). Numbers in columns indicate the number of animals/group.

Then, we assessed the influence of losartan, an AT1 receptor antagonist, on hemorrhage-induced AVP secretion. Similarly to NBQX, both hemorrhage (F_(1,34)_=7.27, P=0.01) and losartan (F_(1,34)_=6.39, P=0.016) administration changed plasma AVP levels. Additionally, interaction between the two factors was statistically significant (F_(1,34)_=14.63, P<0.001). The *post hoc* analysis showed that losartan administration did not significantly change plasma AVP secretion in control rats; however, hemorrhage-induced AVP secretion was abolished in rats submitted to previous *icv* losartan administration (P<0.001) (Figure 3). Therefore, AT1 receptor activation seemed to be necessary for AVP secretion after hemorrhage.

## Discussion

L-glutamate is the main excitatory neurotransmitter in the central nervous system and its receptors are ubiquitously expressed in the brain. Pharmacological studies demonstrated the existence of three main families of ionotropic glutamate receptors: AMPA, kainate, and NMDA receptors. AMPA and kainate receptors are rapidly activated and then desensitized, whilst NMDA receptors are involved in long-term plasticity and higher cognitive functions (15). Magnocellular neurons in the PVN and SON are richly innervated by glutamatergic afferents (16), which suggests a role for L-glutamate on the control of AVP secretion. NMDA receptors are implicated in AVP release in response to dehydration (17), and we have previously demonstrated that after hypertonic extracellular volume expansion (EVE) the increase in plasma AVP is mediated by NMDA receptors, but it is not changed by AMPA receptors whilst opposite results are found for oxytocin (OT) secretion after hypertonic EVE (13). Thus, different glutamate receptors are possibly involved in the control of different functions in magnocellular PVN and SON neurons.

In addition to the participation of different L-glutamate receptors in the control of AVP secretion, in this study we showed that AMPA receptors were involved in the control of AVP release after hemorrhage. Thus, an increase in plasma osmolality activates a glutamatergic circuit, which in turn activates NMDA receptors to induce AVP release (13), while a decrease in blood volume activates a different glutamatergic circuit that activates AMPA receptors to induce AVP release. These results indicated that AMPA and NMDA receptors may differentially regulate AVP release in response to different hydromineral challenges. Previous studies showed that pharmacological blockade of AMPA receptors in the SON attenuates the glutamate-evoked AVP release, while NMDA blockade in the SON does not change AVP release (10). These results indicate that in the SON, L-glutamate activates AMPA receptors to induce AVP release, similar to the results we demonstrated after hemorrhage and contrary to the results we found after hypertonic EVE. However, the same study showed that intra-PVN administration of L-glutamate does not affect AVP release in normal conditions but increases AVP release after pre-treatment with NMDA antagonists, indicating that in the PVN NMDA signaling inhibits L-glutamate induced AVP release (10). This suggests that, although both nuclei mediate AVP release, different physiological stimuli affect different populations of neurons in the SON and PVN to elicit AVP secretion. Together, the findings from this study and others (10,13) indicate the possibility that a decrease in blood volume activates AMPA receptors to increase plasma AVP, whilst increases in osmolality preferentially activate the NMDA receptors to increase plasma AVP. Hemorrhage causes hypotension/hypovolemia without changing osmolality, thus this information is conveyed to the brain mainly through changes in the activity of volume receptors and baroreceptors; whereas, challenges in osmolality are perceived primarily by osmosensitive neurons in specific brain regions, such as circumventricular organs (3). Possibly, these different pathways recruit diverse neural circuitries, neurotransmitters, and receptors reaching different magnocellular neuron populations to mediate the increase in AVP release, thus explaining the different L-glutamate receptors involved in this hormone secretion after hypertonic EVE and hemorrhage.

A decrease in blood volume and/or arterial pressure activates the renin-angiotensin system (RAS), which culminates in the increase of ANG II plasma levels; ANG II is a hormone that acts primarily through AT1 receptors to increase blood pressure (3). In addition to its direct effect on vasoconstriction, ANG II also activates the local RAS of different tissues, such as the brain RAS. The increased activity of the brain RAS enhances the hypertensive effect of the peripheral RAS since it increases the activity of systems that also increase blood pressure, such as the sympathetic activity and AVP secretion (18). Previous studies show that ANG II induces AVP release (6,8,9). Hemorrhage increases ANG II in both the plasma and the hypothalamus (19). In this study, we demonstrated that *icv* losartan blunted hemorrhage-induced AVP secretion, which indicates that brain ANG II-AT1 receptor signaling was necessary for this response. In a study that used a hemorrhage protocol similar to ours (∼25% blood removed) (20), *icv* losartan administration also blunted hemorrhage-induced AVP secretion. However, these results contradict a previous report that described that central losartan administration did not change AVP in response to hemorrhage (19). These differences are possibly due to the different severity of the hemorrhage used since they withdrew a greater volume of blood. Thus, in a moderate blood loss the blockade of AT1 receptors with losartan reduces AVP secretion, but in severe blood loss other additional mechanisms probably sustain AVP-secretion in response to hemorrhage after the blockade of AT1 signaling. Another possible explanation is the pathway used for losartan administration, as they used an intravitreal injection (19). These results highlight the complexity of the regulation of AVP secretion and further indicate that different circuits may be involved in the regulation of AVP release after different stimuli.

Collectively our data showed that hemorrhage required AMPA, but not NMDA, signaling to elicit AVP secretion in response to hemorrhage, indicating that different challenges to body fluid homeostasis may recruit different glutamatergic circuits within the brain that in turn activate different glutamate receptors to induce AVP release. In conclusion, both AMPA and AT1 receptor signaling were necessary for the hemorrhage-induced AVP release, which suggests an important role for ANG II and L-glutamate in AVP release after hypovolemia/hypotension.
